# Integrative Network Analysis of Bioactive Compounds from *Punica granatum* L. Peel: Multi-Target Mechanisms in Wound Healing

**DOI:** 10.5812/ijpr-170416

**Published:** 2026-07-18

**Authors:** Seyyed Shahab Shokri, Ebrahim Salimi Sabour, Seyed Morteza Hosseini, Hossein Reza Shateri

**Affiliations:** 1 Student Research Committee, Baqiyatallah University of Medical Sciences, Tehran, Iran; 2 Department of Pharmacognosy and Traditional Pharmacy, Faculty of Pharmacy, Baqiyatallah University of Medical Sciences, Tehran, Iran; 3 Spiritual Health Research Center, Life Style Institute, Baqiyatallah University of Medical Sciences, Tehran, Iran; 4 Department of Pharmacotherapy, School of Pharmacy, Mashhad University of Medical Sciences, Mashhad, Iran

**Keywords:** Pomegranate, Wound Healing, Network Pharmacology, Focal Adhesion

## Abstract

**Background:**

Wound-healing agents often have limited efficacy and require prolonged recovery times, prompting growing interest in developing herbal-based formulations. Among these, *Punica granatum* L. has attracted considerable attention because of its high polyphenolic content. Despite its widespread use, the precise pharmacological targets underlying its wound-healing effects remain poorly understood and require systematic investigation.

**Objectives:**

This study aimed to elucidate the underlying pharmacological mechanisms of the topical wound-healing properties of *P. granatum* L. using a network pharmacology approach.

**Methods:**

Bioactive compounds of *P. granatum* L. and their potential target genes were identified using the Traditional Chinese Medicine Systems Pharmacology Database and Analysis Platform (TCMSP), Similarity Ensemble Approach (SEA), and SwissTargetPrediction databases. Wound healing-related genes were retrieved from the GeneCards database. Genes intersecting *P. granatum* L. targets and wound healing-associated genes were subjected to functional enrichment analyses, including protein–protein interaction (PPI), Gene Ontology (GO), and Kyoto Encyclopedia of Genes and Genomes (KEGG) pathway analyses. The PPI network was further analyzed using Cytoscape, and the phytoconstituent–target interaction network was visualized using Gephi. These findings were validated using molecular docking.

**Results:**

A total of 40 intersecting genes were identified as potential *P. granatum* L. targets involved in wound healing. Among these, EGFR, PTPN11, HRAS, IGF1R, and ESR1 were identified as key hub genes. Functional enrichment analysis indicated that the most significantly enriched signaling pathways included the MAPK, PI3K-Akt, EGFR tyrosine kinase inhibitor resistance, focal adhesion, and FoxO signaling pathways. Molecular docking analysis confirmed favorable binding of quercetin and ellagic acid to the hub targets EGFR, IGF1R, and ESR1.

**Conclusions:**

These findings elucidate the pharmacological pathways underlying *P. granatum*-mediated wound healing and suggest that *P. granatum* L. acts as a multi-target modulator in the wound-healing process.

## 1. Background

In response to disruption of the normal structure and function of the epidermis and dermis, a multifactorial process known as wound healing occurs to restore tissue integrity (1).

Several conventional therapeutic agents have been used in wound management; however, they often show low efficacy and undesirable outcomes, including scarring and prolonged recovery times. These limitations have motivated the search for more effective wound-healing agents, including diverse wound dressings and plant-derived therapeutics (2). Among these, pomegranate (*Punica granatum* L.), belonging to the Lythraceae family, has attracted increasing interest because of its enriched polyphenolic constituents and its traditionally recognized use in wound healing (3).

*P. granatum* L. peel, which accounts for approximately 30% - 50% of the whole fruit, is often considered agricultural waste (4). Both in vitro and in vivo investigations have demonstrated the wound-healing potential of *P. granatum* L. peel extract (5). However, its underlying molecular targets and wound-healing signaling mechanisms remain largely undefined (6). Its diverse bioactive constituents are associated with several molecular pathways that have largely been studied separately, including oxidative stress modulation, inflammatory cascade inhibition, extracellular matrix remodeling, and cell proliferation signaling (7, 8). Conventional pharmacological approaches, which are often limited by the one compound-one target paradigm, are inadequate to fully explain the polypharmacology and complex molecular relationships underlying the effects of herbal agents, particularly in multifactorial processes such as wound healing, which are driven by multiple interconnected molecular networks and pathways. In this context, network pharmacology, with its systematic multi-component, multi-target paradigm, offers a powerful strategy that uses high-throughput computational techniques to characterize interactions between bioactive compounds and gene targets, thereby identifying hub targets and critical biological pathways. Ultimately, this approach generates mechanistic hypotheses regarding key targets and pathways for drug development, which can then be validated through in vivo experiments (9).

## 2. Objectives

This study aimed to predict and analyze the potential pharmacological mechanisms underlying topical wound healing mediated by *Punica granatum* L. peel using network pharmacology approaches.

These results provide valuable insights into the pharmacological mechanisms of *P. granatum* L. peel and provide a mechanistic framework for the future design of *P. granatum*-based wound-healing formulations.

## 3. Methods

The main databases and web tools used in this study were TCMSP 2.3, PubChem, SEA, SwissTargetPrediction, GeneCards 5.25, Venny 2.1.0, Search Tool for the Retrieval of Interacting Genes/Proteins (STRING) 12.0, and the Database for Annotation, Visualization and Integrated Discovery (DAVID) 2025. Cytoscape 3.10.4 and Gephi 0.10.1 were used for network visualization.

To identify the main bioactive constituents of *Punica granatum* L. peel, the TCMSP database was searched using the term "Shiliupi" (Granati Pericarpium, *P. granatum* L. rind) (10). Because this study focused on the topical wound-healing activity of phytoconstituents, oral pharmacokinetic parameters, such as oral bioavailability and drug-likeness, were not used as restrictive screening criteria. Instead, compounds were selected based on verification of their presence in the peel through an extensive literature review. Irrelevant compounds retrieved from TCMSP were excluded after manual verification.

To mitigate potential incompleteness and limited phytochemical coverage of the TCMSP-derived dataset, a literature-validation strategy was applied. Phytoconstituents not represented in the initial TCMSP list were subsequently supplemented through manual curation of peer-reviewed phytochemical studies retrieved from PubMed using the herb name and the keyword "active components." Given the substantial variability in *P. granatum* L. peel phytochemical composition across different geographical regions, only compounds reported in at least three independent studies were retained for subsequent analysis to improve the reliability and reproducibility of the results (11).

PubChem was used to obtain Simplified Molecular Input Line Entry System (SMILES) chemical notations of phytoconstituents for subsequent analyses.

To identify targets of *P. granatum* L. peel phytoconstituents, SEA (human proteins only, P < 0.01) and SwissTargetPrediction (probability ≥ 0.5) were used by importing the SMILES chemical notation of each phytoconstituent into each database.

Human genes related to wound healing were identified from the GeneCards database using the keyword "wound healing." Targets with GeneCards Inferred Functionality Scores greater than 60 were retained for subsequent analysis.

Intersecting targets between *P. granatum* L. peel phytoconstituents and the disease were identified using the Venny web tool.

The web-based STRING database was used to identify significant clusters of intersecting targets and to visualize the PPI network (organism, *Homo sapiens*; highest confidence score, > 0.900; duplicate targets excluded). The Markov Cluster Algorithm (MCL) clustering method was applied, with the inflation parameter set to 3, to identify relevant clusters within the PPI network. To improve visualization, dotted lines were used as edges.

The PPI data were analyzed to rank nodes using the cytoHubba plugin in Cytoscape 3.10.4. Potential hub genes were defined as the top five ranked nodes using the Maximal Clique Centrality (MCC) method (12).

To annotate the biological functions of intersecting genes and elucidate the roles of target proteins in biological pathways, DAVID 2025 was used to obtain functional enrichment analysis results, including significantly enriched biological processes, molecular functions, cellular components, and KEGG pathways (13). Only terms with P < 0.001 were considered statistically significant.

A phytoconstituent-target network was visualized and analyzed using Gephi 0.10.1, with degree centrality metrics used for node sizing and the Fruchterman-Reingold layout algorithm used for optimal visualization (14).

To provide additional computational validation of compound-target interactions, molecular docking was performed between the two key phytoconstituents, quercetin and ellagic acid, and the three major hub targets, EGFR, IGF1R, and ESR1. The X-ray crystallographic structures of the proteins were downloaded from the RCSB Protein Data Bank (https://www.rcsb.org; accessed May 20, 2026): EGFR (PDB ID: 1M17), IGF1R (PDB ID: 3D94), and ESR1 (PDB ID: 3ERT). Protein preparation was performed in UCSF Chimera 1.17.1 by removing water molecules and co-crystallized ligands, followed by the addition of polar hydrogens and atomic charges. Binding sites were defined according to the position of the original co-crystallized ligand. Ligand structures were obtained from PubChem and prepared using the same protocol. Docking was performed using AutoDock Vina implemented in PyRx 1.0. The coordinates of the docking box centers were set at 21.86, 0.26, and 52.76 for EGFR (1M17); 24.61, 18.14, and -5.52 for IGF1R (3D94); and 31.54, -1.63, and 25.55 for ESR1 (3ERT). A grid box of 20 x 20 x 20 Å was centered on each binding site, and the exhaustiveness parameter was set to 4 (15). For each protein-ligand pair, the top-ranked pose, corresponding to the lowest predicted binding energy, was selected and visualized in BIOVIA Discovery Studio Visualizer 2025 as 3-dimensional binding poses and 2-dimensional interaction diagrams.

To validate the docking protocol, the co-crystallized ligand of each target was extracted and redocked into its original binding site under the same grid box coordinates and docking parameters. Docking accuracy was assessed by calculating the heavy-atom root mean square deviation (RMSD) between the crystallographic ligand pose and the corresponding top-ranked redocked pose.

## 4. Results

A total of 26 phytoconstituents were retrieved from the TCMSP database. After literature-based validation, 21 compounds were retained for downstream analysis, including ellagic acid, methyl gallate, gallic acid (3,4,5-trihydroxybenzoic acid), gallic acid-3-O-(6'-O-galloyl)glucoside, catechin, quercetin, isoquercetin (hirsutrin), kaempferol, luteolin, luteolin-7-O-glycoside, astragalin, punicalagin, punicalin, corilagin, casuarinin, granatin B, beta-sitosterol, oleanolic acid, ursolic acid, beta-peltatin, and pelletierine. Compounds not reported as naturally occurring in the peel of *Punica granatum* L., including fritillaziebinol, 1-methyl-3-isopropoxy cyclohexane, 09762_FLUKA, officinalisin, and mannitol, were excluded based on previous phytochemical reports.

Given the incomplete compound coverage of TCMSP and the well-documented importance of polyphenols in *P. granatum* L. peel, key literature-supported polyphenolic compounds were added to the phytoconstituent list, including p-coumaric acid, ferulic acid, cinnamic acid, chlorogenic acid, syringic acid, caffeic acid, and 2,3-dimethoxybenzoic acid, which were classified as phenolic acids (4, 16). Rutin, naringenin, apigenin, and epicatechin were obtained from the literature as frequently reported flavonoids (17, 18). Regardless of geographic origin, cyanidin, delphinidin, and pelargonidin glucoside derivatives were selected as predominant anthocyanins in *P. granatum* L. peel (Table S1 in Supplementary File) (16).

Protein target identification for these 32 bioactive components using two distinct databases, SEA and SwissTargetPrediction, yielded 681 targets (Table S2 in Supplementary File). Additionally, 91 wound-healing-related targets were obtained from the GeneCards database (Table S3 in Supplementary File).

**Figure 1.**
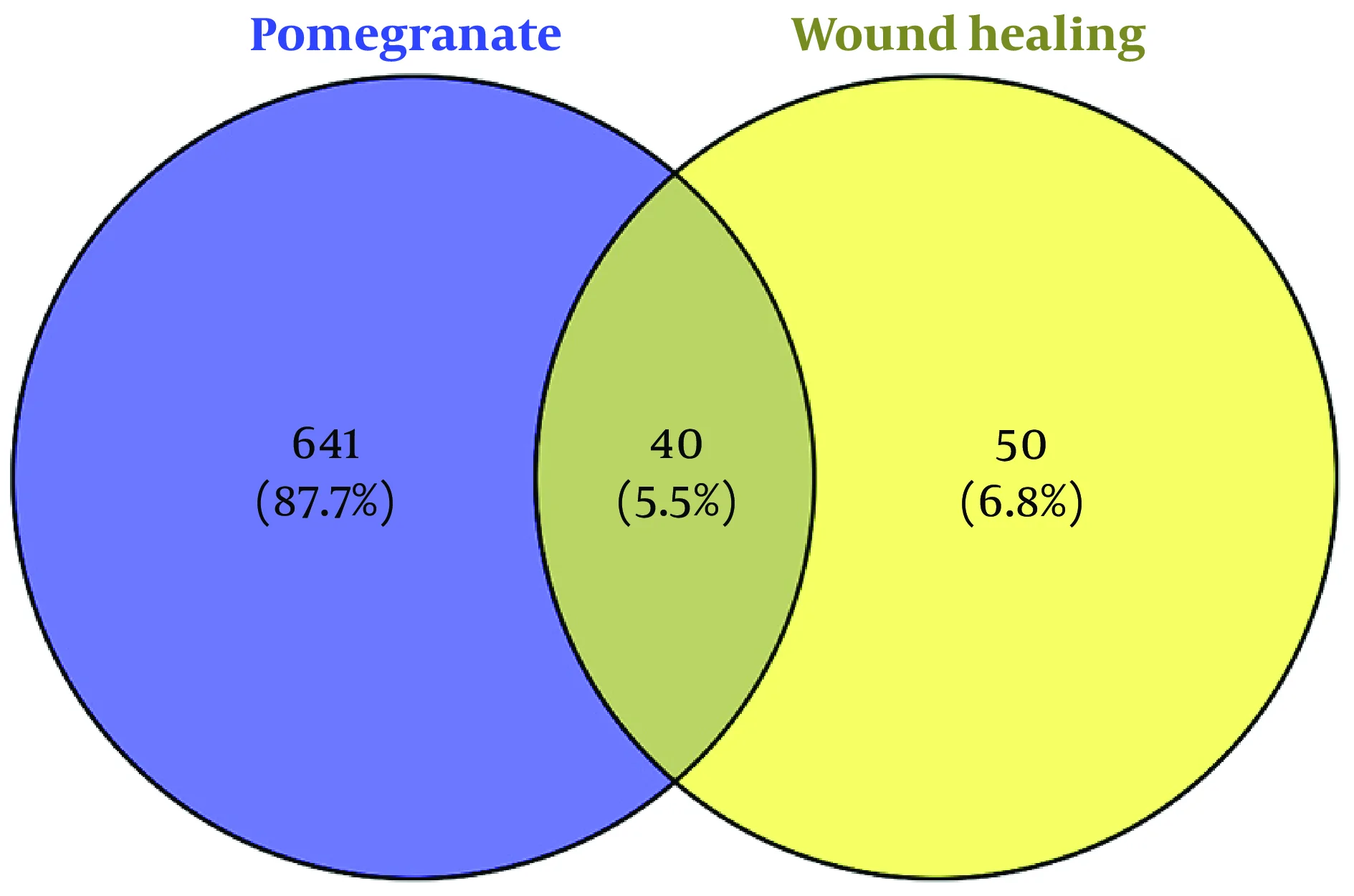
Venn diagram illustrating the intersection between gene targets of *P. granatum* L. phytoconstituents and target genes associated with the wound healing process.

Forty targets were identified as intersecting target genes between *P. granatum* L. phytoconstituents and wound healing. The Venn diagram is shown in Figure 1. These intersecting genes were considered potential target genes of *P. granatum* L. involved in the wound-healing process.

### 4.1. Protein-Protein Interaction Network Construction and Topology Analysis

**Figure 2.**
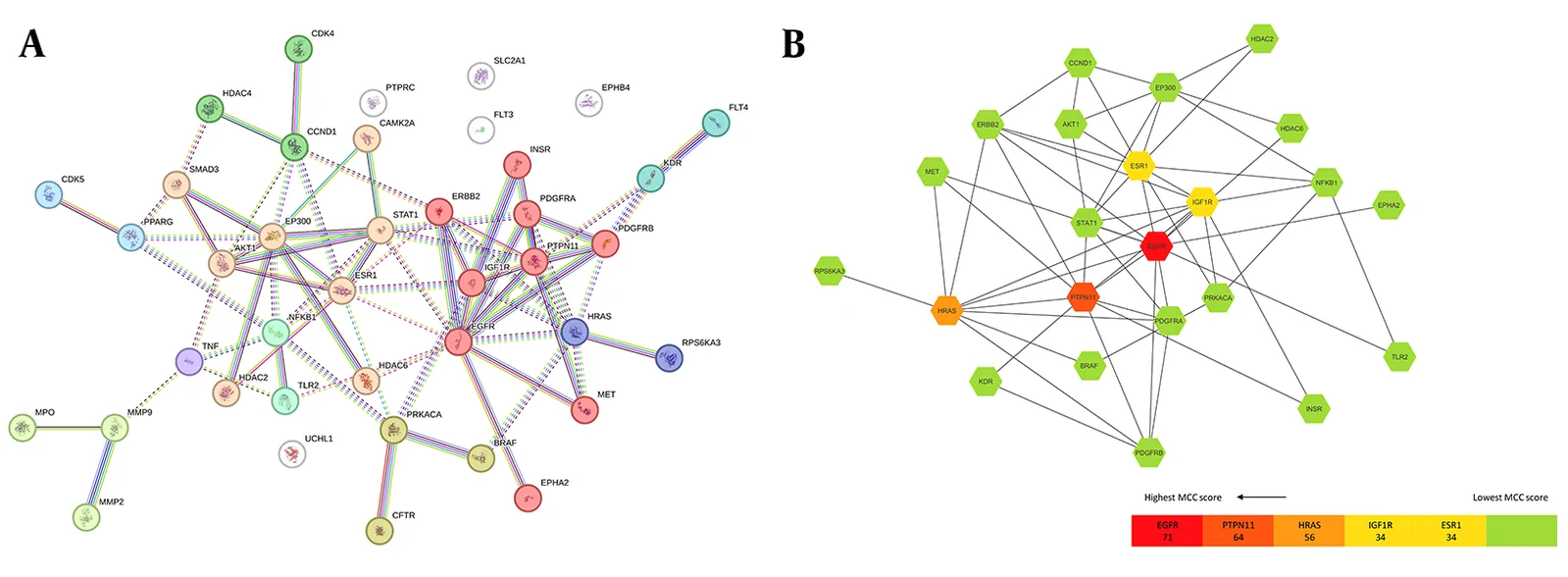
A, Protein-protein interaction (PPI) network of the overlapping genes between *P. granatum* L. phytoconstituent-predicted targets and wound healing-related genes. The PPI network was retrieved from the STRING database. Clustering was performed using the MCL algorithm; discrete clusters and dotted connectivity indicate functionally related protein modules. B, Key hub genes within the STRING-derived PPI network of overlapping genes between predicted targets of *P. granatum* L. compounds and wound healing-related genes. Key hub genes were identified using the cytoHubba plugin (MCC method). Node color indicates MCC score from red (highest hub centrality) to yellow (lower).

The PPI network is widely used to identify interactions among protein targets in the context of complex processes. The STRING PPI network comprised 40 nodes and 75 edges and is visualized in Figure 2A.

Given the complexity of the original network obtained from the STRING database, the PPI data were imported into Cytoscape to explore the importance of potential targets within the protein network and to identify the main cluster within the network. The cytoHubba plugin was used to identify hub genes. The top five ranked nodes identified by cytoHubba were EGFR, PTPN11, HRAS, IGF1R, and ESR1, representing the hub genes within the PPI network (Figure 2B).

Key hub genes identified through cytoHubba MCC analysis are highlighted within the PPI network. A color gradient ranging from red (highest MCC score, greatest hub centrality) to yellow (lower MCC score) indicates the relative importance of each hub gene within the network. The legend provides a reference scale for interpreting centrality ranks.

**Figure 3.**
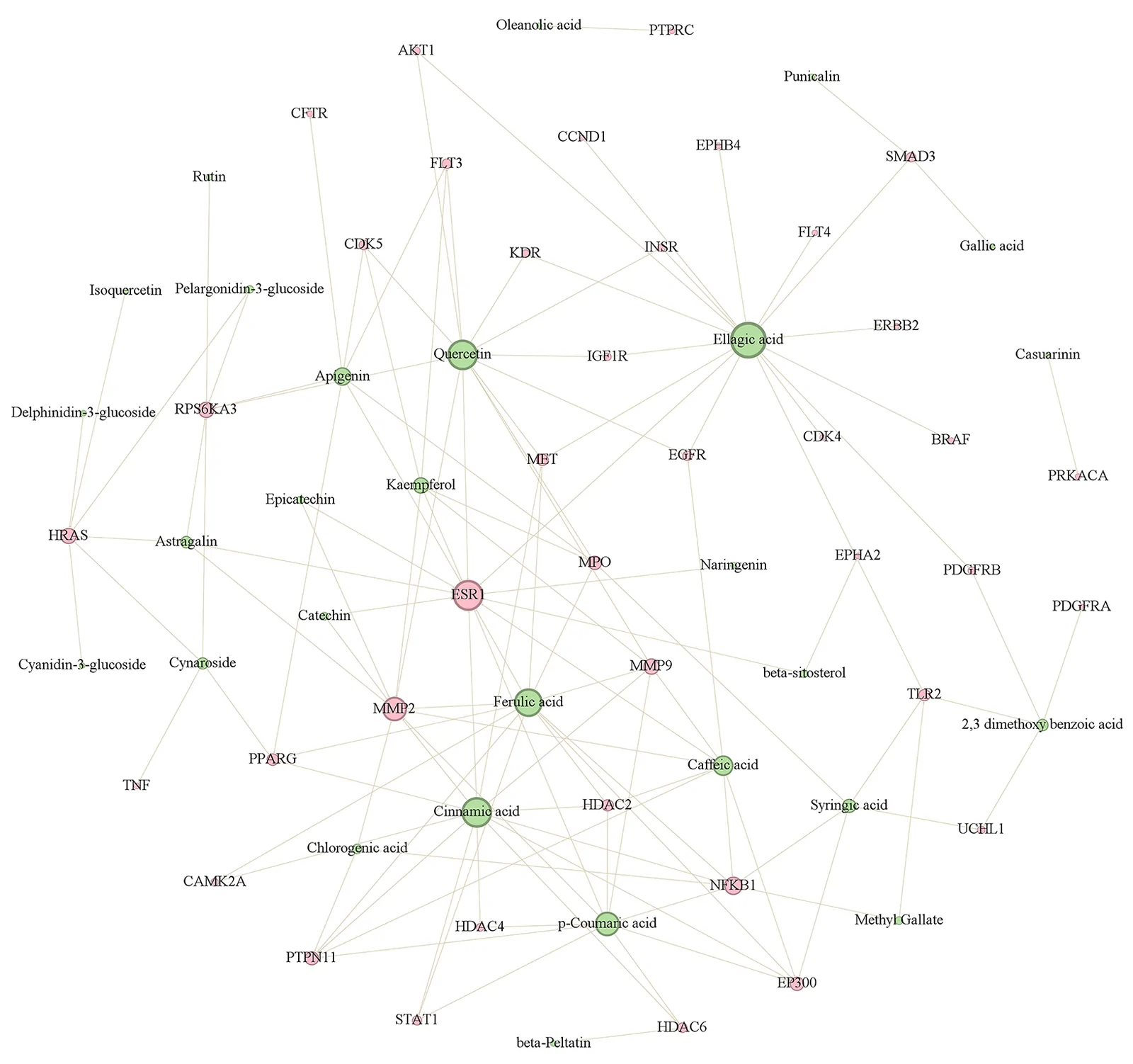
Phytoconstituent-target interaction network. The network illustrates interactions between *P. granatum* L. phytochemical compounds and wound-healing-associated genes generated using Gephi. Nodes represent compounds (green) and genes (pink). Node size is proportional to interaction connectivity; larger nodes correspond to higher degree values within the network.

The *P. granatum* L. bioactive component-target network was constructed using Gephi (Figure 3; 67 nodes and 125 edges).

**Figure 4.**
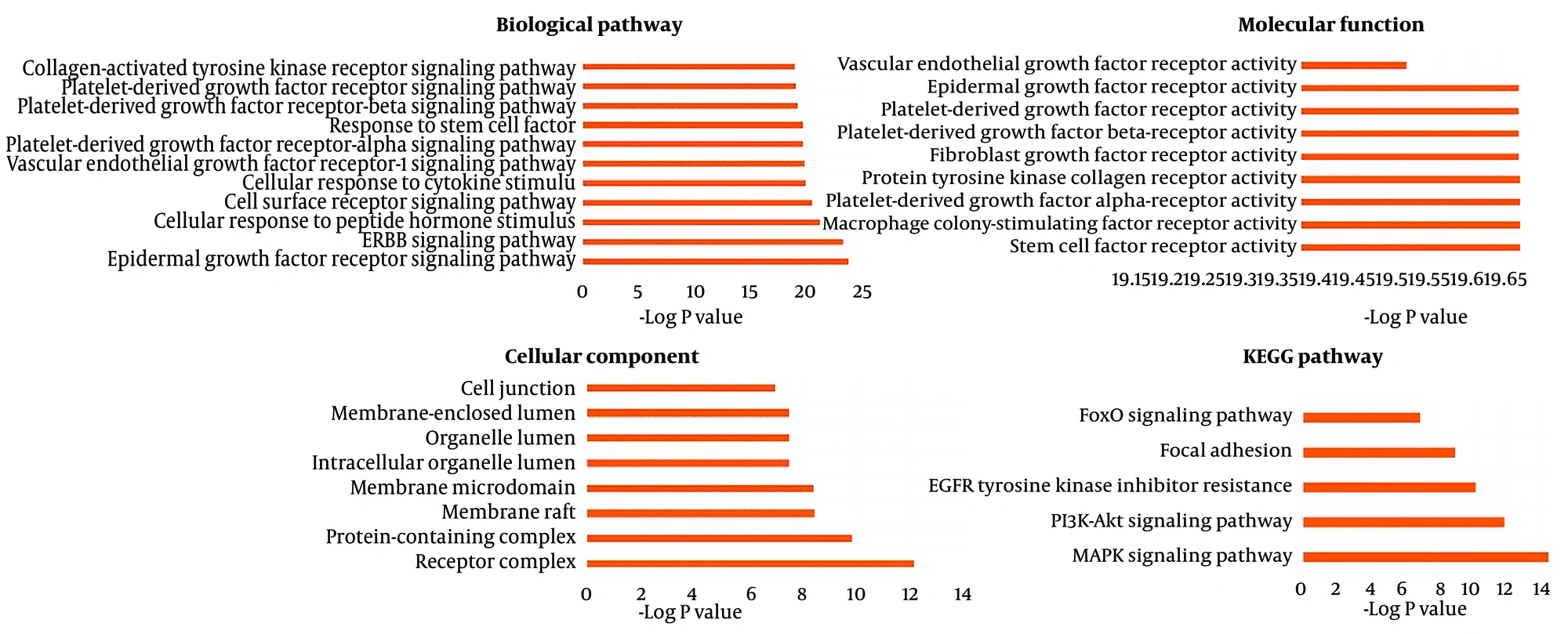
Functional enrichment analysis of wound-healing-related gene targets influenced by *P. granatum* L. phytoconstituents.

To further explore the underlying mechanisms of *P. granatum* L. peel as a wound-healing agent, GO enrichment analysis was performed using the 40 potential target genes of *P. granatum* L. involved in the wound-healing process (Figure 4).

Bar charts depict the results of GO enrichment across biological process, molecular function, and cellular component categories, as well as the KEGG pathway enrichment profile. Data were analyzed using DAVID 2025 and visualized in Microsoft Excel. Terms are ranked by the -log(P) value, highlighting processes associated with cell proliferation, migration, and growth-factor signaling.

GO enrichment analysis was performed in three categories: biological process, molecular function, and cellular component. Among the 537 enriched biological processes with P < 0.001, the most relevant were EGFR signaling, ephrin receptor signaling pathway, ERBB signaling pathway, cellular response to peptide hormone stimulus, cell surface receptor signaling pathway, cellular response to cytokine stimulus, VEGFR-1 signaling pathway, PDGF-alpha/beta signaling pathway, and response to stem cell factor. In the molecular function category, 10 significantly enriched and biologically relevant functions were protein tyrosine kinase collagen receptor activity, PDGF-alpha/beta receptor activity, stem cell factor receptor activity, macrophage colony-stimulating factor receptor activity, PDGF receptor activity, EGFR activity, fibroblast growth factor receptor activity, insulin-like growth factor receptor activity, VEGF receptor activity, and transmembrane ephrin receptor activity.

For the cellular component category, 40 cellular components were retrieved from DAVID (P < 0.001), of which the top eight were receptor complex, protein-containing complex, membrane raft, membrane microdomain, organelle lumen, intracellular organelle lumen, membrane-enclosed organelle, and cell junction.

KEGG pathway enrichment yielded 74 pathways with P < 0.001. Among these, the five most relevant KEGG pathways are shown in Figure 4, including mitogen-activated protein kinase signaling pathway, phosphatidylinositol 3-kinase-AKT signaling pathway, EGFR tyrosine kinase inhibitor resistance, focal adhesion, and Forkhead Box O signaling pathway.

**Figure 5.**
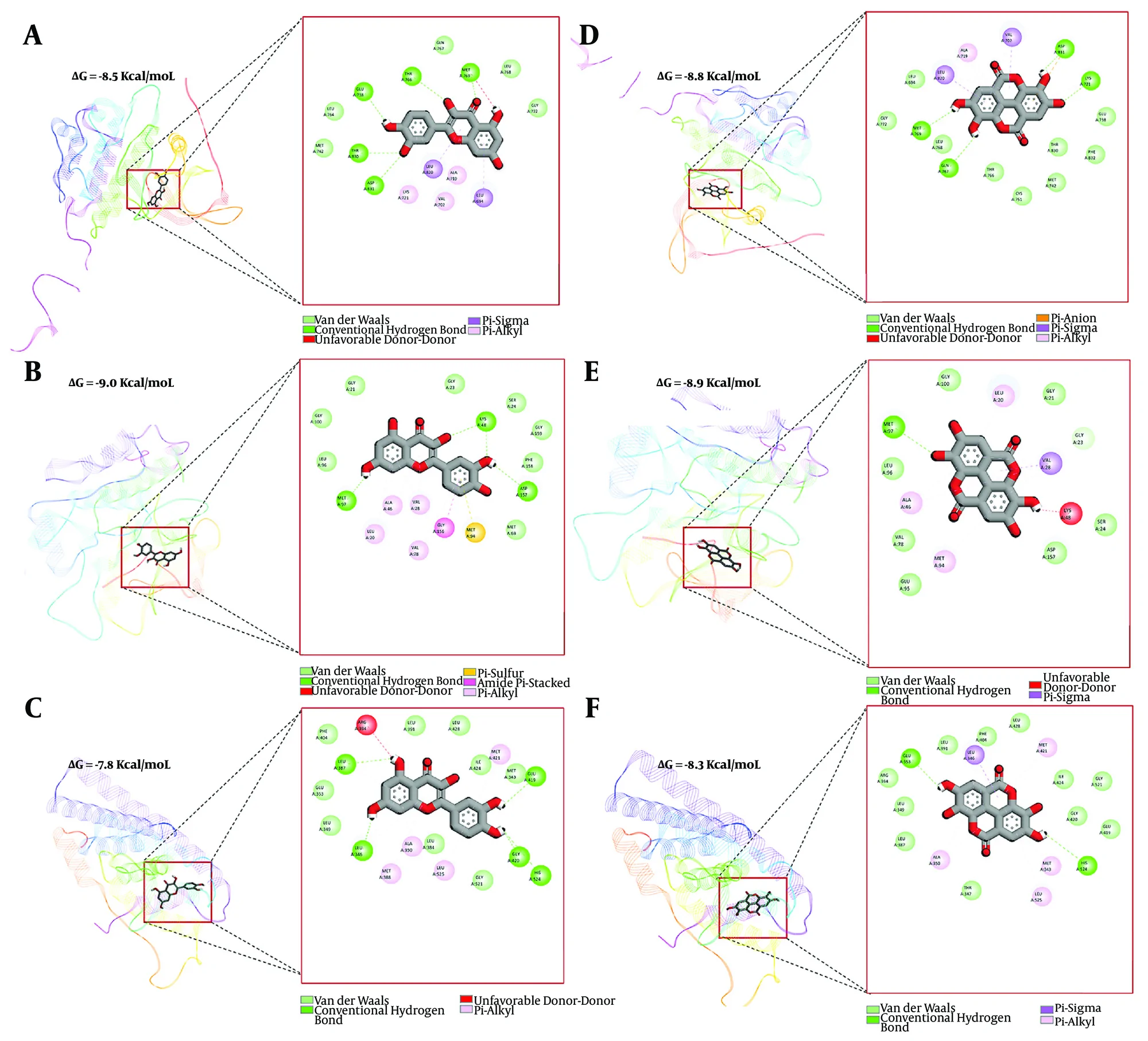
Representative 3-dimensional binding poses (left in each panel) and the corresponding 2-dimensional protein-ligand interaction maps (right in each panel) are shown for each protein-ligand pair. A-C, Quercetin docked to EGFR (Delta G = -8.5 kcal/mol), IGF1R (Delta G = -9.0 kcal/mol), and ESR1 (Delta G = -7.8 kcal/mol), respectively. D-F, Ellagic acid docked to EGFR (Delta G = -8.8 kcal/mol), IGF1R (Delta G = -8.9 kcal/mol), and ESR1 (Delta G = -8.3 kcal/mol), respectively. Predicted interactions include hydrogen bonding and van der Waals contacts, with additional hydrophobic/pi-related contacts depending on the receptor cavity. Binding energies (Delta G, kcal/mol) are reported as estimated docking scores for the selected top-ranked poses.

Molecular docking further supported the interaction of quercetin and ellagic acid with the hub targets EGFR, IGF1R, and ESR1, as indicated by favorable binding energies (Delta G ≤ -7 kcal/mol) in all docked poses. Quercetin showed binding energies of -8.5 kcal/mol for EGFR, -9.0 kcal/mol for IGF1R, and -7.8 kcal/mol for ESR1. Within the EGFR binding pocket, quercetin formed hydrogen bonds with MET769, GLU738, THR830, ASP831, and THR766, along with one unfavorable donor-donor interaction (Figure 5A). In IGF1R, it formed four hydrogen bonds with ASP157, MET97, and LYS48 (Figure 5B), whereas in ESR1, hydrogen bond interactions were observed with LEU387, LEU346, GLY420, HIS524, and GLU419, accompanied by one unfavorable donor-donor interaction (Figure 5C). Similarly, ellagic acid demonstrated strong binding to all three proteins. It formed hydrogen bonds with LYS721, ASP831, MET769, and GLN767 in EGFR, along with van der Waals, conformational hydrogen bond, conformational donor-donor, Pi-Sigma, and Pi-Alkyl interactions (Figure 5D). In IGF1R, it established a hydrogen bond with MET97, with the same set of additional interactions (Figure 5E), and in ESR1, it formed hydrogen bonds with GLU353 and HIS524, with similar supplementary interactions (Figure 5F).

These results provide structural validation for the network-based predictions and support the role of ellagic acid and quercetin as key active constituents influencing EGFR, IGF1R, and ESR1 signaling in wound healing.

To validate the docking protocol, the co-crystallized ligand of each target protein was redocked into its native binding site using the same docking parameters. The heavy-atom RMSD values between the crystallographic and redocked ligand poses were 2.372 Å for EGFR (1M17), 0.838 Å for IGF1R (3D94), and 1.581 Å for ESR1 (3ERT), demonstrating acceptable to excellent reproduction of the experimental binding modes. The corresponding binding affinities of the co-crystallized ligands were -7.0, -14.1, and -9.8 kcal/mol, respectively.

## 5. Discussion

The present integrative bioinformatic analysis was conducted to elucidate the potential mechanistic network underlying the wound-healing activity of *Punica granatum* L. using integrative computational approaches. Previous in vivo and preclinical studies have shown the wound-healing potential of pomegranate peel and fruit extracts, demonstrating effects such as upregulation of VEGF, TGF-beta1, collagen, and nitric oxide synthesis, as well as antimicrobial and antioxidant effects (19, 20). Despite these pharmacological findings, the underlying target genes and molecular mechanisms remain poorly characterized. Considering that the peel constitutes nearly 30% - 50% of the fruit and is often discarded as waste, exploring its potential gene-level therapeutic value in wound healing is of growing interest.

The present study suggests that *P. granatum* L. peel may exert wound-healing activity through the coordinated regulation of several growth factor-related pathways. GO enrichment highlighted modulation of molecular functions and biological processes associated with tissue regeneration, including EGFR, FGFR, VEGF, and PDGF signaling, as well as ephrin-mediated cell communication, which is involved in cellular migration and extracellular matrix synthesis (21, 22). These predicted interactions occur mainly within receptor complexes, cellular membranes, and junctional regions, reflecting precise control over proliferation and remodeling processes (23).

Consistently, KEGG enrichment identified MAPK, PI3K-Akt, FoxO, and focal adhesion pathways as predicted principal mediators of cell survival, migration, and stress adaptation (24). Network analysis further identified HRAS, EGFR, PTPN11, IGF1R, and ESR1 as potential hub genes regulating cellular proliferation, cross-talk, and migration signaling (25, 26). Collectively, these findings predict that pomegranate peel phytoconstituents act through multi-target mechanisms integrating growth-factor responsiveness and cytoskeletal remodeling, which could contribute to wound repair processes.

Our network-based approach revealed that ellagic acid and quercetin were among the major phytoconstituents that may potentially exert prominent topical wound-healing activity by interacting with key signaling targets such as EGFR, HRAS, IGF1R, PTPN11, and ESR1.

Additionally, molecular docking provided structural support for the compound-target interactions predicted by the network pharmacology analysis. Quercetin and ellagic acid demonstrated favorable binding affinities toward the three hub proteins EGFR, IGF1R, and ESR1, forming stable networks of hydrogen bonds, van der Waals interactions, and hydrophobic contacts. The docking conformations indicated that these phytoconstituents occupy the receptor binding sites, thereby supporting their predicted regulatory influence on EGFR-, IGF1R-, and ESR1-mediated pathways. Compared with the co-crystallized ligands, quercetin and ellagic acid exhibited comparable predicted binding affinities toward the investigated targets. These findings suggest that both compounds can adopt stable binding conformations within the binding sites of the target proteins, thereby strengthening the mechanistic interpretation that *P. granatum* L. peel exerts wound-healing effects through multi-target engagement involving growth factor signaling, proliferation, and matrix remodeling.

Similar wound-healing activities have been reported for several medicinal plants and their bioactive derivatives. For instance, *Aloe vera* (L.) Burm.f. and *Centella asiatica* (L.) Urb. enhance re-epithelialization and collagen deposition, whereas *Calendula officinalis* L. and *Camellia sinensis* (L.) Kuntze contribute to proangiogenic and anti-inflammatory effects. Consistent with these findings, other plant-derived agents, such as carvacrol, have demonstrated significant potential to modulate fibroblast activity, collagen synthesis, and antimicrobial defense. Collectively, these reports, along with the results for *Curcuma longa* L. and our findings on *P. granatum*, reinforce the therapeutic relevance of plant-based multi-target approaches in tissue regeneration and wound healing (2, 27). In light of these findings, future pharmaceutical formulations may prioritize *P. granatum* L. varieties and regional sources with higher concentrations of ellagic acid and quercetin to enhance bioactive potency and clinical outcomes.

It should be noted that the present investigation is based on an in silico analysis; further in vitro and in vivo experiments are required to validate the predicted pharmacological mechanisms and fully confirm the therapeutic potential of *P. granatum* L. peel extract.

### 5.1. Conclusions

The enriched data demonstrated that *Punica granatum* L. peel phytoconstituents, especially ellagic acid and quercetin, may modulate several signaling cascades, such as MAPK, PI3K-AKT, and focal adhesion pathways, potentially contributing to enhanced cellular proliferation, migration, and tissue regeneration. Moreover, hub genes such as EGFR, HRAS, IGF1R, PTPN11, and ESR1 were identified as central regulators within the wound-healing PPI network, revealing the molecular mechanisms underlying the pharmacological potential of *P. granatum* L. peel. These insights support a systems-pharmacology view of *P. granatum* L. peel as a multifunctional wound-healing agent and suggest that cultivars rich in ellagic acid and quercetin represent promising subjects for further experimental validation in topical formulations.

ijpr-25-1-170416-s001.zip

## Data Availability

The data presented in this study are uploaded as a supplementary file during submission and are openly available for readers upon request. The Excel file (.xlsx) contains four sheets, named: Supplementary table S1, Supplementary table S2, Supplementary table S3, Supplementary table S4.
